# Supplementary material for: Impact of Red Imported Fire Ant Nest-Building on Soil Properties and Bacterial Communities in Different Habitats
Source: Animals (Basel). 2023 Jun 18;13(12):2026. doi: 10.3390/ani13122026 (PMC10294795; doi:10.3390/ani13122026)
Supplement: Supplementary file 1 [file animals-13-02026-s001.zip › animals-2410903-supplementary.pdf]

ID	domain	phylum	class	order	family	genus	species	unclassified	total ASVs
TS-N_1	24	12	74	395	693	2161	157	1	3517
TS-N_2	19	20	80	437	773	2424	184	1	3938
TS-N_3	36	20	84	434	673	2299	178	1	3725
TS-CK_1	10	13	45	382	641	2583	102	0	3776
TS-CK_2	13	13	35	314	594	2405	112	1	3487
TS-CK_3	19	14	54	347	713	2769	140	0	4056
BS-N_1	6	6	51	112	438	1831	184	0	2628
BS-N_2	8	8	54	110	482	1990	201	0	2853
BS-N_3	6	4	53	137	455	1982	222	0	2859
BS-CK_1	3	8	36	76	327	1355	143	0	1948
BS-CK__2		2	12	30	61	314	1439	124	1982
BS-CK__3		7	12	38	85	375	1378	141	2037
LS-N_1	33	20	106	320	779	2241	178	0	3677
LS-N_2	12	11	61	237	542	1776	147	0	2786
LS-N_3	39	28	95	318	716	1972	146	1	3315
LS-CK_1	35	24	67	345	602	2189	95	1	3358
LS-CK_2	43	21	91	467	701	2788	109	0	4220
LS-CK_3	49	24	87	371	799	2657	139	1	4127
FS-N_1	19	6	86	415	592	1935	181	0	3234
FS-N_2	6	6	77	419	546	1996	175	0	3225
FS-N_3	27	9	70	366	562	1862	171	0	3067
FS-CK_1	29	14	63	467	737	1995	112	0	3417
FS-CK_2	23	9	50	480	768	1928	124	0	3382
FS-CK_3	30	14	51	461	690	1884	138	0	3268
RS-N_1	20	9	66	375	606	1765	135	1	2977
RS-N_2	21	11	81	434	799	2105	144	1	3596
RS-N_3	22	5	72	399	806	2054	141	1	3500
RS-CK_1	21	11	60	441	473	1705	65	1	2777
RS-CK_2	23	11	56	379	459	1637	63	3	2631
RS-CK_3	19	6	45	428	455	1636	90	2	2681
